# Comparative study of Amsel’s criteria and Nugent scoring for diagnosis of bacterial vaginosis in a tertiary care hospital, Nepal

**DOI:** 10.1186/s12879-021-06562-1

**Published:** 2021-08-17

**Authors:** Rajshree Bhujel, Shyam Kumar Mishra, Santosh Kumar Yadav, Kesang Diki Bista, Keshab Parajuli

**Affiliations:** 1 Department of Clinical Microbiology, Institute of Medicine, Tribhuvan University Teaching Hospital, Kathmandu, Nepal; 2 Department of Microbiology, Rajarshi Janak University, Janakpurdham, Nepal; 3 Department of Obstetrics and Gynecology, Institute of Medicine, Tribhuvan University Teaching Hospital, Kathmandu, Nepal

**Keywords:** Amsel’s criteria, Bacterial vaginosis, Nugent scoring, Vaginal discharge

## Abstract

**Background:**

The most common pathological cause of abnormal vaginal discharge in reproductive-aged women is bacterial vaginosis (BV). Amsel’s criteria and Nugent scoring systems are commonly employed approaches for the diagnosis of BV. Despite the Nugent scoring system being the gold standard method for diagnosing BV, Amsel’s criteria are generally preferred in clinical setup owing to the fact Nugent scoring requires considerable time and expert microscopist. This study was conducted to determine the diagnostic value of Amsel’s criteria by comparing it with the Nugent scoring system.

**Methods:**

This was a descriptive cross-sectional study conducted at Tribhuvan University Teaching Hospital, Nepal from October 2016 to September 2017. Vaginal specimens were collected from a total of 141 women presenting with abnormal vaginal discharge. The sensitivity, specificity, positive predictive value, and negative predictive value of Amsel’s criteria were calculated, and each component of Amsel’s criteria was compared to the Nugent scoring system.

**Results:**

The sensitivity, specificity, positive predictive value, and negative predictive value of Amsel’s criteria were 50%, 98.2%, 87.5%, and 88.8% respectively. The clue cells showed 100% specificity and vaginal discharge with pH > 4.5 had 89.3% sensitivity while compared with Nugent’s scoring system.

**Conclusions:**

Amsel’s criteria can be used as an adjunct method to Nugent scoring for the diagnosis of BV in the hands of skilled manpower in resources limited countries. The presence of clue cell and positive whiff test of Amsel’s criteria shows good match with Nugent’s score.

**Supplementary Information:**

The online version contains supplementary material available at 10.1186/s12879-021-06562-1.

## Background

The vagina has a metabolically and microbiologically complex environment [1]. In a reproductive age group woman, the healthy vagina should have an abundance of Gram-positive rods, usually *Lactobacillus* spp, a low pH (≤ 4.5), and the absence of both facultative and obligatory anaerobic Gram-negative bacilli [1]. There are many infectious and non-infectious causes of vaginal environment disturbance. Among the pathological causes, bacterial vaginosis (BV) is the commonest reproductive tract infection of reproductive age group women [2]. BV occurs due to alteration of vaginal flora in which normal flora (*Lactobacilli*) is replaced by a mixed bacterial flora which includes *Gardnerella vaginalis*, *Mobiluncus* species, *Mycoplasma hominis*, *Bacteroides* species, and some other anaerobic bacteria [2]. It is characterized by inflammation of vaginal mucosa together with clinical symptoms of abnormal vaginal discharge, itching, burning sensation, and discomfort [1].

BV is more prevalent in developing countries than in developed countries [3]. The prevalence of BV in developing countries ranges from 20 to 47% among non-pregnant women [4] which depends on geographical location, socio-economic status, and ethnicity [5]. Although the prevalence of BV is highest in most African, and lowest in Asian and European countries in general, some parts of Africa have low, and Asia and Europe have higher rates of BV [5]. Centers for Disease Control and Prevention (CDC) has reported a 29.2 % BV rate in American women of the age group 14–49 years [6]. In Nepal, few studies have been conducted about BV but there are no published reports on the diagnostic value of Amsel’s criteria for the diagnosis of BV. Manandhar et al. [7] and Bhargava et al. [8] have found 2.5 and 54.3% BV cases among non-pregnant Nepalese women.

BV has several adverse effects such as amniotic fluid infection, i.e., chorioamnionitis, premature rupture of membranes, low-birth weight, premature birth, increased incidence of pelvic infection after abortion, vaginal cuff cellulitis after hysterectomy, endometritis, cervicitis, urinary tract infection, cervical intraepithelial neoplasia, increased probability of ectopic pregnancy, infertility, and chronic pelvic pain [9]. Therefore, diagnosis and treatment of BV in time is of great importance.

The most common method for the diagnosis of BV is the clinical criteria described by Amsel et al. [10] and microscopic criteria developed by Nugent et al. [11]. Nugent scoring system is a gold standard method due to its reproducibility and high sensitivity but is time-consuming, costly, and needs laboratory equipment and specialists which create great problem in developing countries with limited resources as in Nepal. On the other hand, Amsel’s criteria are rapid, inexpensive, and simple, thus one should know the sensitivity and specificity of Amsel’s criteria in comparison to the Nugent scoring system. In the light of the above considerations, this study was designed to find out the diagnostic value of Amsel’s criteria by comparing it with the Nugent scoring system for the diagnosis of BV.

## Methods

### Study design and setting

A descriptive cross-sectional study was conducted at Tribhuvan University Teaching Hospital (TUTH) from October 2016 to September 2017 (12 months period). Vaginal samples were collected at the Gynecology Outpatient Department (GOPD) and processed for necessary investigations in the clinical microbiology laboratory. A total of 141 High Vaginal Swab (HVS) specimens were collected from the patients who presented with abnormal Per Vaginal (PV) discharge and fulfilled inclusion criteria. Informed written consent was taken before enrolment in the study. Per speculum examination was performed by a Gynecologist and the vaginal mucosa was inspected for the presence of erythema, lesions, and discharge. The posterior fornix was swabbed with two sterile swabs from each female by the Gynecologist. After collection of HVS, color, consistency, and homogeneity of the specimen were noted, and the swabs were placed in a sterile container with a drop of sterile normal saline, and further processed in microbiology laboratory.

### Inclusion criteria

All sexually active women in the reproductive age group who visited GOPD with a history of abnormal PV discharge were included in the study.

### Exclusion criteria

Patients with bleeding per vagina, patients with genital tract malignancies (cervical and endometrial carcinomas and vulval), patients under treatment while presenting to GOPD, and pregnant women.

### Laboratory investigation

The first swab was used for pH measurement, wet mount preparation, and whiff test. Similarly, the second swab was processed for Gram’s staining. Vaginal pH was determined by using litmus paper; the change in the color of litmus paper was observed and noted. The wet mount preparation was carried out for clue cells. The whiff test was done by adding few drops of 10% potassium hydroxide (KOH) solution to the discharge on a clean glass slide and noted for a strong fishy odor for positive results. For the diagnosis of BV, Amsel’s criteria were followed. According to Amsel’s criteria, if at least three of the following four criteria were fulfilled, it was confirmed to be a case of BV [10].

- Grayish white, thin, and homogeneous vaginal discharge,
- Vaginal pH higher than 4.5,
- Fishy or amine odor after addition of 10% KOH, and.
- Presence of clue cell (> 20%) on microscopic examination.

Gram staining was done to determine the Nugent’s score by a clinical microbiologist. Nugent’s score system was based on the number of different morphotypes of bacteria, viz., *Lactobacillus*-like (large uniform Gram-positive bacilli), *Gardnerella vaginalis*-like (small pleomorphic Gram-variable bacilli), or *Prevotella*/*Bacteroides*-like (small Gram-negative bacilli), and *Mobiluncus*-like (curved Gram-variable bacilli). A Nugent score of 7–10 was interpreted as consistent with BV and a score of 4–6 as intermediate, while a score of 0–3 was interpreted as negative for BV [11].

### Statistical analysis

Data were analyzed using SPSS 16.0 version and Microsoft Excel Sheet and interpreted according to frequency distribution and percentage. A chi-square test was used to determine significant association wherever applicable with a *p*-value of less than 0.05 regarded as significant.

## Results

Among a total of 141 cases, the prevalence of BV was found to be 11.3% (n = 16) based on Amsel’s criteria and 19.9% (n = 28) from Nugent’s score criteria. The present study revealed maximum BV cases belonging to the age group 25–34 years followed by 15–24 years. Out of 28 cases reported positive from Nugent’s score system, 14 cases were positive from Amsel’s criteria too. Two more cases were positive by Amsel’s criteria but negative according to Nugent’s score system. While comparing Amsel’s criteria with Nugent’s score which is a gold standard method for the diagnosis of BV, sensitivity, specificity, positive predictive value and negative predictive value of Amsel’s criteria were 50%, 98.2%, 87.5%, and 88.8%, respectively (Table 1).

**Table 1:** Comparison of Amsel’s criteria with Nugent’s score as a gold standard for the diagnosis of BV

Methods of diagnosis		Nugent’s criteria			Sensitivity (%)	Specificity (%)	PPV (%)	NPV (%)
		Positive	Negative	Total				
Amsel’s criteria	Positive	14	2	16	50.0	98.2	87.5	88.8
	Negative	14	111	125				
	Total	28	113	141				

In our study, statistical results showed both Amsel’s criteria and Nugent’s score system was statistically significant (*p* < 0.001) in the chi-square test (Table 2).

**Table 2:** Comparison between the result of Amsel’s criteria and Nugent’s score for the diagnosis of BV

Diagnostic criteria		Results of Nugent’s score			*p-*value
		BV (%)	No BV (%)	Total (%)	
Results of Amsel’s criteria	BV (%)	14 (9.9)	2 (1.4)	16 (11.3)	< 0.001
	No BV (%)	14 (9.9)	111 (78.7)	125 (88.7)	
	Total (%)	28 (19.8)	113 (80.1)	141 (100)	

BV, bacterial vaginosis

Among 141 samples, the frequency of Amsel’s criteria finding was as follows: 114 had pH > 4.5 followed by thin white homogenous discharge in 81 cases, positive whiff test in 20, and clue cells seen in 13 cases under microscopic examination (Additional file 1). Each individual test method was compared with Nugent’s score system and various diagnostic measures were calculated including sensitivity, specificity, positive predictive value, and negative predictive value (Table 3).

**Table 3:** Comparison of various diagnostic methods of Amsel’s criteria with Nugent’s score as a gold standard for the diagnosis of BV

Diagnostic methods	Positive	Negative	Sensitivity (%)	Specificity (%)	PPV (%)	NPV (%)
Thin white homogenous discharge	81	60	60.7	43.4	21.0	81.7
pH > 4.5	114	27	89.3	21.2	21.9	88.9
Positive whiff test	20	121	35.7	91.2	50.0	85.1
Presence of Clue cell	13	128	46.4	100	100	88.3

The current study showed the presence of clue cell as the individual Amsel’s criterion has 100% specificity. In addition, a positive whiff test has the second-highest specificity (91.2%) with the lowest sensitivity (35.7%). On the other hand, vaginal discharge with pH > 4.5 has the highest sensitivity (89.3%) with the lowest specificity (21.2%), and thin white homogenous discharge has 60.7% sensitivity and 43.4% specificity (Table 3).

Our study showed that the presence of clue cells and the positive whiff test were highly suggestive of BV (P < 0.001) (Table 4).

**Table 4:** Comparison of various diagnostic methods of Amsel’s criteria with Nugent’s score for the diagnosis of BV

Diagnostic methods of Amsel’s criteria	Number	Nugent’s score		*p*-value
		BV (%)	No BV (%)	
Thin white homogenous discharge	81	17 (21.0)	64 (79.0)	0.33
pH > 4.5	114	25 (21.9)	89 (78.1)	0.20
Positive Whiff test	20	10 (50.0)	10 (50.0)	< 0.001
Clue cell seen	13	13 (100)	0 (0)	< 0.001

BV, bacterial vaginosis

## Discussion

The classical diagnostic methods like Amsel’s criteria and Nugent’s scoring systems remain the most feasible and economical options for the diagnosis of BV especially in developing countries where multiple criteria are used for the confirmation of BV [11, 12]. For Amsel’s criteria, clinical diagnosis and a few simple laboratory tests are used, whereas Nugent’s criteria involves assessment of normal flora in the Gram-stained smear of vaginal discharge. Although culture is regarded as the gold standard approach for diagnosing many bacterial infections, it cannot be a good method for diagnosing BV, since the bacteria that cause BV are difficult to isolate and these type of organisms are also present in small number as normal vaginal flora [13].

In addition to scientific deliberations, choosing an appropriate method for laboratory diagnosis requires consideration of convolution, cost, and the frequency of uninterpretable specimens. Some alternative definitive diagnostic methods, such as polymerase chain reaction (PCR), nucleic acid hybridization test, and proline aminopeptidase activity have been developed but they are not cost-effective [14]. Therefore, choosing the right diagnostic method for BV is important. Accurate diagnosis of BV is challenging because the sensitivity and specificity of most of the available methods do not constitute a greater advantage over the classical diagnostic methods.

The current study showed the prevalence of BV based on Amsel’s criteria was 11.3% and Nugent’s scoring criteria was 19.9% in a total of 141 samples. These findings are consistent with the study of Udayalaxmi et al. [13] and Rao et al. [14]. But the results of the current study contrast with some other studies [4, 15, 16] in that they have reported more cases of BV by Amsel’s criteria than Nugent’s scoring system. This variation in result might be due to the demanding nature for the genuine diagnosis of BV, the distinction in the accessibility of these two tests, and the intricacy of the laboratory diagnostic method.

The sensitivity, specificity, positive predictive value, and negative predictive value of Amsel’s criteria was 50.0%, 98.2%, 87.5%, and 88.8%, respectively while comparing with Nugent’s score as found in our study. A similar type of result was documented by Modak et al. [17] and Taj et al. [18]. But studies conducted by Udayalaxmi et al. [13], Mohammadzadeh et al. [19], and Hossien et al. [16] showed high sensitivity of Amsel’s criteria while comparing with the current study. According to Baverly et al. [20], the variability in clinician interpretation is an important factor. Although the diagnosis of BV by Amsel’s criteria is simple but is relatively insensitive. Since Gram staining is a reproducible and reliable method for the diagnosis of BV, the results obtained using Amsel’s criteria must be verified using Gram staining.

According to Amsel’s criteria, we performed multiple tests for the diagnosis of BV. Our study compared each individual test of Amsel’s criteria with Nugent’s scoring system, so the presence of clue cells indicated 100% specificity with 46.4% sensitivity. The result of the present study is similar to that of Bansal et al. [21], who also showed the highest specificity with low sensitivity of clue cells out of four diagnostic methods of Amsel’s criteria. But studies conducted by Mengistie et al. [22], Mohammadzadeh et al. [19], and Hossien et al. [16] revealed clue cells have higher sensitivity than specificity. Low sensitivity for the identification of clue cells may depend upon the ability of the microbiologists to analyze wet mount microscopy.

In the present study, vaginal discharge with pH > 4.5 had the highest sensitivity (89.3%) with the lowest specificity (21.2%) which is similar to the result of Bansal et al. [21], Simoes et al. [23], and Gautam et al. [24] but different from Mengistie et al. [22]. It may be due to the fact that the pH is easily affected by the presence of blood or semen in the vaginal sample or the application of lubricant gel during sexual intercourse. Cervical mucus itself has a pH of 6.0 which may interfere with vaginal pH. The whiff test in current study showed 91.2% specificity with the lowest sensitivity (35.7%). This result is different from Bansal et al. [21] and Mohammadzadeh et al. [19]. This difference in the sensitivity of the whiff test may be ascribed to the personalized nature of the test due to the sensation ability of the person doing the test. The other factor may be either the absence or presence of a low amount of amine-producing microorganisms.

## Conclusions

Amsel’s criteria showed high specificity and low sensitivity as compared to Nugent’s score. Among the criteria used, presence of clue cells showed 100% specificity for the diagnosis of BV and vaginal discharge with pH > 4.5 had the best sensitivity. Diagnosis of BV by Amsel’s criteria can be easily done with the limited facility, and are thus applicable in setups where there is a dearth of laboratory facilities. The presence of clue cells can be used as a diagnostic criterion combined with the whiff test to assist in diagnosing BV in the outpatient clinics. Thus, Amsel’s criteria can be used as an adjunct method to the Nugent scoring system for the diagnosis of BV in the hands of skilled manpower in resources limited countries.

## Limitation of the study

Culture for anaerobic bacteria and specific pathogens causing BV were not done. Asymptomatic cases were missed.

## Supplementary Information

**Additional file 1: Photograph S1.**Clue cell in wet mount preparation (400X magnification). **Photograph S2.** Clue cell in Gram stain (1000X magnification).

## Data Availability

The datasets used and/or analysed during the current study are available from the corresponding author on reasonable request.

## Abbreviations

BV: Bacterial Vaginosis
CDC: Centers for Disease Control and Prevention
GOPD: Gynecology Outpatient Department
HVS: High Vaginal Swab
KOH: Potassium Hydroxide
PV: Per Vaginal
PCR: Polymerase Chain Reaction
PPV: Positive Predictive Value
NPV: Negative Predictive Value
SPSS: Statistical Package for the Social Sciences
TUTH: Tribhuvan University Teaching Hospital
