# Supplementary material for: Comparative study of Amsel’s criteria and Nugent scoring for diagnosis of bacterial vaginosis in a tertiary care hospital, Nepal
Source: BMC Infect Dis. 2021 Aug 17;21:825. doi: 10.1186/s12879-021-06562-1 (PMC8369704; doi:10.1186/s12879-021-06562-1)
Supplement: Supplementary file 1 — Additional file 1: Photograph S1.Clue cell in wet mount preparation (400X magnification). Photograph S2. Clue cell in Gram stain (1000X magnification). [file 12879_2021_6562_MOESM1_ESM.docx]

**Photographs**


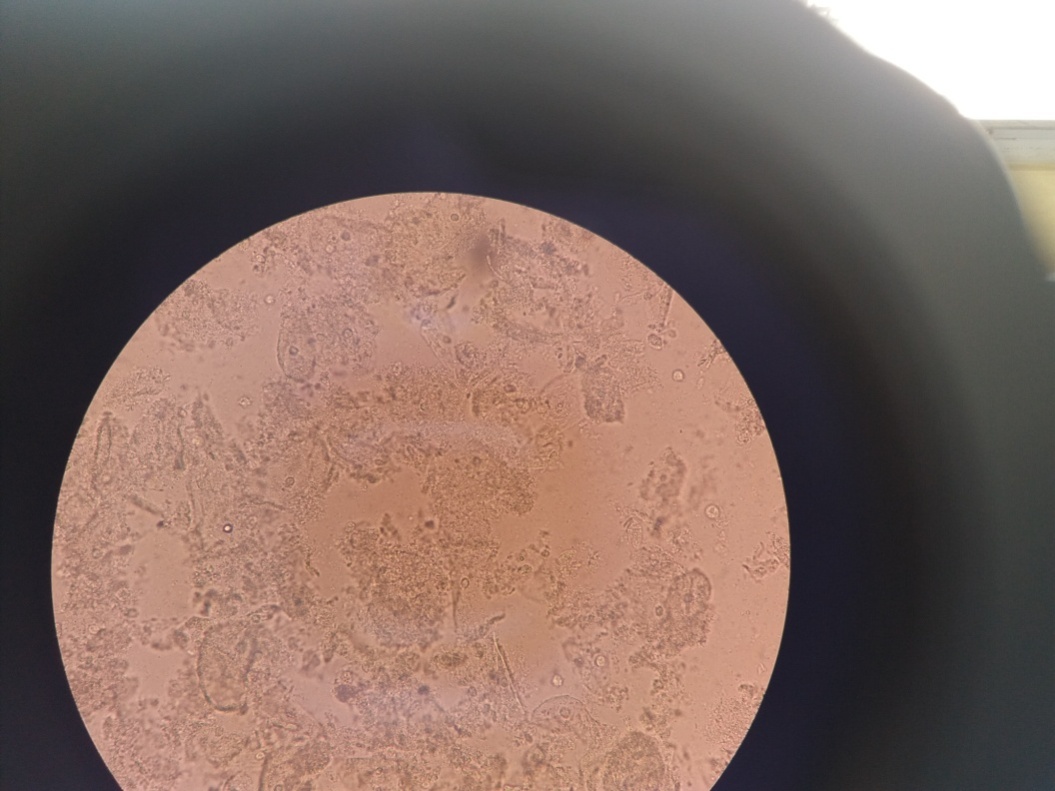


**Photograph S1** Clue cell in wet mount preparation (400X magnification)


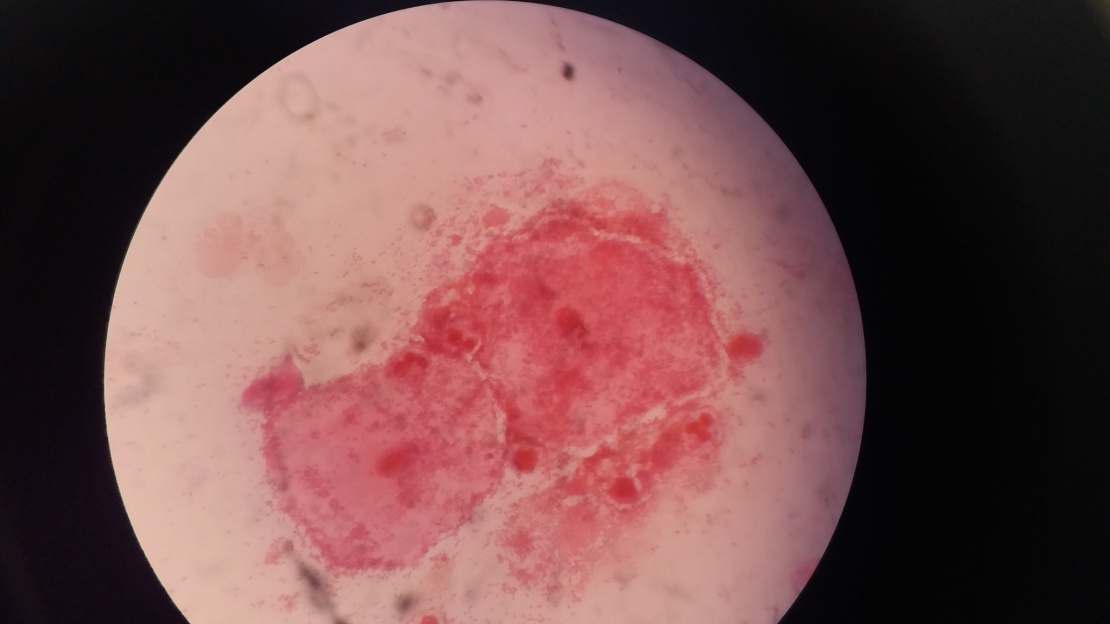


**Photograph S2** Clue cell in Gram stain (1000X magnification)
